# Associations of maternal pre-pregnancy body mass index with pregnancy complications and infant adiposity at 6 months corrected age: a two-center retrospective cohort study

**DOI:** 10.3389/fnut.2026.1820970

**Published:** 2026-06-26

**Authors:** Rong Zeng, Peng Yuan, Daisheng Zhang, Qiang Zhang, Ying Lei

**Affiliations:** 1 Chengdu Wenjiang Maternal and Child Health Hospital, Chengdu, China; 2 Bazhong Maternal and Child Health Hospital, Bazhong, Sichuan, China; 3 Department of Gynaecology and Obstetrics, Chengdu Women’s and Children’s Central Hospital, School of Medicine, University of Electronic Science and Technology of China, Chengdu, China

**Keywords:** breastfeeding, gestational diabetes mellitus, gestational hypertensive disorders, infant growth, infant obesity, maternal obesity, pre-pregnancy BMI

## Abstract

**Objective:**

To assess associations of maternal pre-pregnancy BMI with pregnancy complications, adverse perinatal outcomes, and infant adiposity at 6 months corrected age.

**Methods:**

This two-center retrospective cohort included 707 mother-infant dyads from 790 eligible pregnancies between March 2021 and June 2023. Pre-pregnancy BMI was classified as underweight (<18.5 kg/m^2^), normal-weight (18.5–23.9 kg/m^2^), overweight (24.0–27.9 kg/m^2^), and obesity (≥28.0 kg/m^2^). Infant anthropometry at 6 months corrected age was converted to WHO z-scores. Multivariable logistic regression was used to explore factors associated with adverse perinatal outcomes and infant obesity at 6 months corrected age.

**Results:**

Among 707 women, 47 (6.6%) were underweight, 314 (44.4%) normal-weight, 220 (31.1%) overweight, and 126 (17.8%) obese. Higher BMI category was associated with higher HbA1c, gestational diabetes mellitus, gestational hypertensive disorders, postpartum hemorrhage, macrosomia, NICU admission, and infant adiposity z-scores. Compared with normal weight, overweight (adjusted OR 1.38, 95% CI 1.01–1.90) and obesity (adjusted OR 1.86, 95% CI 1.24–2.78) were associated with higher odds of adverse perinatal outcomes. Maternal obesity was associated with higher odds of infant obesity at 6 months corrected age (adjusted OR 2.23, 95% CI 1.18–4.20), whereas breastfeeding was associated with lower odds (adjusted OR 0.65, 95% CI 0.39–0.94).

**Conclusion:**

Maternal pre-pregnancy overweight and obesity were associated with pregnancy complications, adverse perinatal outcomes, and increased infant adiposity at 6 months corrected age. Preconception weight management and breastfeeding support may help reduce early metabolic risk.

## Background

The global prevalence of overweight and obesity has risen substantially over recent decades, posing a major public health burden for women of reproductive age. According to the World Health Organization, adult obesity has more than doubled since 1990; in 2022, 2.5 billion adults were overweight, including 890 million adults living with obesity (1). In parallel, maternal overweight and obesity have become increasingly common in obstetric populations and are associated with a broad spectrum of maternal, obstetric, and neonatal complications (2–8).

Accumulating evidence shows that higher pre-pregnancy body mass index (BMI) is associated with increased risks of gestational diabetes mellitus (GDM), gestational hypertensive disorders (GHD), fetal overgrowth, macrosomia, cesarean delivery, and neonatal morbidity (9–15). Maternal adiposity may affect pregnancy progression and fetal growth through multiple interrelated mechanisms, including insulin resistance, chronic low-grade inflammation, endothelial dysfunction, altered placental nutrient transport, and fetal hyperinsulinemia (16–22). Through these pathways, elevated maternal BMI may contribute not only to pregnancy complications but also to altered fetal growth patterns and early postnatal metabolic risk.

The potential influence of maternal obesity may extend beyond the perinatal period. Within the developmental origins of health and disease framework, exposure to an obesogenic intrauterine environment may alter fetal nutrient handling, endocrine regulation, and adiposity trajectories, thereby increasing susceptibility to obesity, insulin resistance, and metabolic disorders later in life (23). Although much of the existing literature has focused on childhood or adolescent outcomes, differences in infant anthropometric profiles may already emerge within the first months after birth, suggesting that early infancy is a clinically relevant window for evaluating the metabolic consequences of maternal pre-pregnancy adiposity.

Gestational weight gain represents another modifiable factor linking maternal BMI with fetal and infant growth. Current guidelines recommend BMI-specific ranges for gestational weight gain, with lower recommended gains for women with overweight or obesity (24, 25). However, appropriate or lower gestational weight gain may not fully offset the metabolic risk associated with elevated pre-pregnancy BMI. In addition, postnatal exposures, particularly breastfeeding, may further modify early-life obesity risk (26–28). Evaluating maternal pre-pregnancy BMI together with gestational weight gain and early feeding practices may therefore provide a more comprehensive understanding of infant growth and adiposity development.

Therefore, we conducted a two-center retrospective cohort study to examine the associations of maternal pre-pregnancy BMI categories with pregnancy complications, adverse perinatal outcomes, and infant growth and obesity at 6 months corrected age.

## Materials and methods

### Study design and participants

This retrospective two-center cohort study was conducted at Chengdu Wenjiang Maternal and Child Health Hospital and Bazhong Maternal and Child Health Hospital between March 2021 and June 2023. Eligible participants were mother-infant dyads who delivered at the participating hospitals and had available pediatric follow-up records. A total of 790 mother-infant dyads who delivered and received pediatric follow-up care met the initial eligibility criteria. We excluded 26 women with multiple pregnancies, 50 women with pre-existing chronic diseases, and 7 pregnancies complicated by major fetal malformations. After exclusions, 707 mother-infant dyads were included in the final analytic cohort.

The study was conducted in accordance with the Declaration of Helsinki. Written informed consent for the use of routinely collected clinical data for research was obtained from participants during hospital care. As this was a retrospective analysis of existing clinical and pediatric follow-up records, no additional intervention was performed. Ethical approval was granted by the Institutional Review Board of Bazhong Maternal and Child Health Hospital (No. 2024478). The study was registered in the Chinese Clinical Trial Registry (ChiCTR2400094710).

### Exposure definition

Pre-pregnancy BMI was calculated as pre-pregnancy weight in kilograms divided by height in meters squared. Pre-pregnancy weight was self-reported at the first prenatal visit, and height was measured and recorded in the medical record. According to Chinese adult BMI criteria, participants were categorized into four groups: underweight group (BMI < 18.5 kg/m^2^), normal-weight group (BMI 18.5–23.9 kg/m^2^), overweight group (BMI 24.0–27.9 kg/m^2^), and obesity group (BMI ≥ 28.0 kg/m^2^). The normal-weight group was used as the reference group in regression analyses.

### Maternal, obstetric, and neonatal variables

Maternal data were extracted from electronic medical records, including maternal age, pre-pregnancy BMI, gravidity, parity, mode of conception, smoking history, alcohol consumption history, reproductive health issues, HbA1c, and gestational weight gain. Mode of conception was classified as natural conception or assisted reproductive technology. Gestational weight gain was calculated as the difference between maternal weight at delivery and pre-pregnancy weight.

Obstetric and neonatal variables included gestational age at birth, preterm birth, premature rupture of membranes, mode of delivery, postpartum hemorrhage, neonatal sex, birthweight, macrosomia, fetal asphyxia, and neonatal intensive care unit (NICU) admission. Macrosomia was defined as birthweight ≥4000 g.

### Pregnancy complications and study outcomes

Pregnancy complications were diagnosed according to nationally recognized clinical guidelines and were recorded in the medical charts. These complications included GDM, ICP, GHD, and fetal growth restriction (FGR) (29–31). Adverse perinatal outcomes were defined as fetal asphyxia and/or NICU admission.

### Infant follow-up at 6 months corrected age

Infants were followed up at 6 months corrected age during routine pediatric visits. Anthropometric measurements were obtained and converted into World Health Organization growth standard z-scores, including weight-for-height z-score (WHZ), weight-for-age z-score (WAZ), height-for-age z-score (HAZ), and BMI-for-age z-score (BAZ) (32). Infant obesity at 6 months corrected age was defined according to BMI-for-age z-score criteria.

Breastfeeding was defined as any breastfeeding recorded at the 6-month corrected-age follow-up visit, including exclusive or partial breastfeeding. Infants receiving no breast milk at the follow-up visit were classified as not breastfed.

### Statistical analysis

Continuous variables were summarized as mean ± standard deviation or median with interquartile range, as appropriate. Categorical variables were presented as number and percentage. Comparisons among the four pre-pregnancy BMI categories were performed using one-way analysis of variance for approximately normally distributed continuous variables, Kruskal-Wallis test for non-normally distributed continuous variables, and chi-square test or Fisher exact test for categorical variables, as appropriate.

Multivariable logistic regression models were constructed to evaluate factors associated with adverse perinatal outcomes and infant obesity at 6 months corrected age. Candidate variables were selected based on clinical relevance and their potential association with the outcome. Odds ratios (ORs) were reported with 95% confidence intervals (CIs). *P*-values for logistic regression models were calculated using Wald tests on the log odds ratio scale. Model calibration was assessed using the Hosmer-Lemeshow goodness-of-fit test, and model discrimination was evaluated using the area under the receiver operating characteristic curve (AUC). A two-sided *P*-value < 0.05 was considered statistically significant. All analyses were performed using IBM SPSS Statistics version 26.0 (Armonk, NY, USA).

## Results

### Study population

**FIGURE 1:**
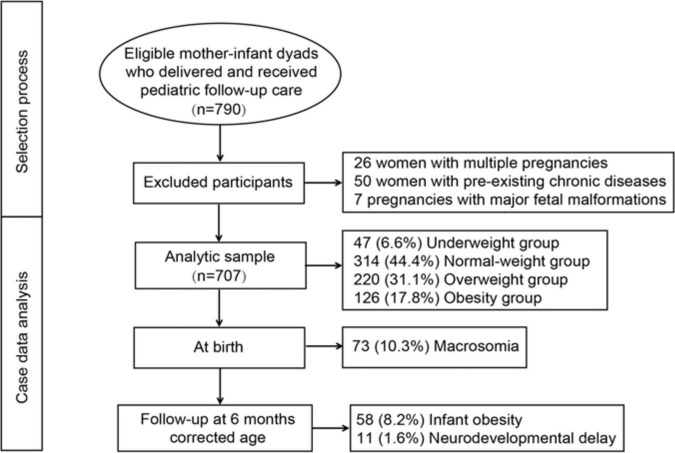
Flow chart of participant selection and analytic cohort.

A total of 790 mother-infant dyads who delivered and received pediatric follow-up care were initially eligible. After excluding 26 women with multiple pregnancies, 50 women with pre-existing chronic diseases, and 7 pregnancies complicated by major fetal malformations, 707 dyads were included in the final analytic cohort (Figure 1).

Among the 707 women, 47 (6.6%) were classified as underweight, 314 (44.4%) as normal-weight, 220 (31.1%) as overweight, and 126 (17.8%) as obese. The mean maternal age was 26.98 ± 4.56 years, and the mean pre-pregnancy BMI was 24.17 ± 3.67 kg/m^2^. Overall, 47 infants (6.6%) were born preterm, 73 infants (10.3%) had macrosomia, and 58 infants (8.2%) met criteria for obesity at 6 months corrected age. Breastfeeding at 6 months corrected age was recorded in 536 infants (75.8%) (Table 1).

**TABLE 1:** Baseline characteristics of the analytic cohort.

Variables	Total
Mother-infant dyads	707
Maternal age, years	26.98 ± 4.56
Pre-pregnancy BMI, kg/m^2^	24.17 ± 3.67
Pre-pregnancy BMI category	
Underweight group, BMI < 18.5 kg/m^2^	47 (6.6%)
Normal-weight group, BMI 18.5–23.9 kg/m^2^	314 (44.4%)
Overweight group, BMI 24.0–27.9 kg/m^2^	220 (31.1%)
Obesity group, BMI ≥ 28.0 kg/m^2^	126 (17.8%)
Smoking	19 (2.7%)
Alcohol consumption history	72 (10.2%)
Natural conception	681 (96.3%)
Assisted reproductive technology	26 (3.7%)
Gestational weight gain, kg	11.80 ± 2.16
Gestational age, weeks	39.72 ± 1.34
Preterm birth	47 (6.6%)
Reproductive health issues	95 (13.4%)
Cesarean section	374 (52.9%)
Male sex	354 (50.1%)
Birthweight, g	3411.09 ± 425.51
Breastfeeding at 6 months corrected age	536 (75.8%)

BMI, body mass index.

### Maternal characteristics by pre-pregnancy BMI category

Maternal age, smoking history, alcohol consumption history, gestational age, preterm birth, gravidity, and parity did not differ significantly across the four BMI categories. Assisted reproductive technology conception was more frequent in women with higher pre-pregnancy BMI (0.0%, 2.2%, 4.5%, and 7.1% in the underweight, normal-weight, overweight, and obesity groups, respectively; *P* = 0.039). Gestational weight gain differed significantly across BMI categories and was lower in women with higher pre-pregnancy BMI (13.05 ± 2.15 kg, 12.34 ± 2.19 kg, 11.30 ± 2.01 kg, and 10.88 ± 1.73 kg, respectively; *P* < 0.001).

Reproductive health issues were more frequent in women with higher BMI (4.3%, 9.9%, 15.9%, and 21.4% across the four groups; *P* = 0.002). HbA1c levels increased across BMI categories, from 4.62 ± 0.68 in the underweight group to 4.85 ± 0.77 in the normal-weight group, 5.37 ± 0.84 in the overweight group, and 5.98 ± 0.89 in the obesity group (*P* < 0.001) (Table 2).

**TABLE 2:** Maternal and neonatal characteristics according to pre-pregnancy BMI category.

Variables	Underweight group	Normal-weight group	Overweight group	Obesity group	*P*-value
Mothers	*N* = 47	*N* = 314	*N* = 220	*N* = 126	
Age, years	25.95 ± 4.25	26.76 ± 4.45	27.18 ± 4.63	27.56 ± 4.75	0.133^a^
Pre-pregnancy BMI, kg/m^2^	17.75 ± 0.55	22.20 ± 1.55	25.39 ± 2.17	29.37 ± 2.57	<0.001^a^
Smoking	1 (2.1%)	8 (2.5%)	6 (2.7%)	4 (3.2%)	0.978^d^
Alcohol consumption history	4 (8.5%)	34 (10.8%)	24 (10.9%)	10 (7.9%)	0.775^d^
Assisted reproductive technology	0 (0.0%)	7 (2.2%)	10 (4.5%)	9 (7.1%)	0.039^d^
Gestational weight gain, kg	13.05 ± 2.15	12.34 ± 2.19	11.30 ± 2.01	10.88 ± 1.73	<0.001^a^
Gestational age, weeks	39.58 ± 1.45	39.86 ± 1.30	39.67 ± 1.33	39.52 ± 1.42	0.072^a^
Preterm birth	5 (10.6%)	18 (5.7%)	15 (6.8%)	9 (7.1%)	0.639^c^
Gravidity	2 (1)	2 (1)	2 (1)	3 (1)	0.341^b^
Parity	0 (1)	0 (1)	0 (1)	0 (1)	0.494^b^
Reproductive health issues	2 (4.3%)	31 (9.9%)	35 (15.9%)	27 (21.4%)	0.002^d^
HbA1c	4.62 ± 0.68	4.85 ± 0.77	5.37 ± 0.84	5.98 ± 0.89	<0.001^a^
GDM	1 (2.1%)	20 (6.4%)	27 (12.3%)	24 (19.0%)	<0.001^d^
ICP	0 (0%)	6 (1.9%)	6 (2.7%)	5 (4.0%)	0.511^d^
GHD	1 (2.1%)	19 (6.1%)	29 (13.2%)	24 (19.0%)	<0.001^d^
FGR	7 (14.9%)	18 (5.7%)	16 (7.3%)	10 (7.9%)	0.153^c^
Premature rupture of membranes	7 (14.9%)	33 (10.5%)	33 (15.0%)	23 (18.3%)	0.150^c^
Cesarean section	20 (42.6%)	160 (51.0%)	121 (55.0%)	73 (57.9%)	0.244^c^
Postpartum hemorrhage	2 (4.3%)	17 (5.4%)	26 (11.8%)	23 (18.3%)	<0.001^d^
Infants					
Male sex	23 (48.9%)	154 (49.0%)	113 (51.4%)	64 (50.8%)	0.954^c^
Birthweight, g	3090.00 ± 380.00	3318.17 ± 380.00	3496.32 ± 419.21	3613.64 ± 432.55	<0.001^a^
Macrosomia	0 (0.0%)	24 (7.6%)	29 (13.2%)	20 (15.9%)	0.003^d^
Fetal asphyxia	1 (2.1%)	2 (0.6%)	3 (1.4%)	3 (2.4%)	0.470^d^
NICU admission	7 (14.9%)	22 (7.0%)	25 (11.4%)	22 (17.5%)	0.010^c^
Breastfeeding at 6 months corrected age	38 (80.9%)	246 (78.3%)	163 (74.1%)	89 (70.6%)	0.267^c^

BMI, body mass index; ART, assisted reproductive technology; GDM, gestational diabetes mellitus; ICP, intrahepatic cholestasis of pregnancy; GHD, gestational hypertensive disorders; FGR, fetal growth restriction; NICU, neonatal intensive care unit.

^a^Average and standard deviation. One-way analysis of variance.

^b^Median (interquartile range). Kruskal-Wallis test.

^c^Number (percentage). Chi-squared test.

^d^Number (percentage). Fisher exact test.

### Pregnancy complications and delivery outcomes

The incidence of GDM increased across BMI categories, with rates of 2.1%, 6.4%, 12.3%, and 19.0% in the underweight, normal-weight, overweight, and obesity groups, respectively (*P* < 0.001). A similar pattern was observed for GHD, with rates of 2.1%, 6.1%, 13.2%, and 19.0%, respectively (*P* < 0.001). In contrast, the rates of ICP did not differ significantly across BMI categories (0.0%, 1.9%, 2.7%, and 4.0%, respectively; *P* = 0.511).

Fetal growth restriction was most frequent in the underweight group, with rates of 14.9%, 5.7%, 7.3%, and 7.9% across the four groups, although the difference was not statistically significant (*P* = 0.153). Postpartum hemorrhage was more frequent in women with higher pre-pregnancy BMI, increasing from 4.3% in the underweight group and 5.4% in the normal-weight group to 11.8% in the overweight group and 18.3% in the obesity group (*P* < 0.001). Premature rupture of membranes and cesarean delivery did not differ significantly across BMI categories (Table 2).

### Neonatal outcomes at birth

Birthweight increased across maternal BMI categories, with mean birthweights of 3090.00 ± 380.00 g, 3318.17 ± 380.00 g, 3496.32 ± 419.21 g, and 3613.64 ± 432.55 g in the underweight, normal-weight, overweight, and obesity groups, respectively (*P* < 0.001). The prevalence of macrosomia also increased across BMI categories, from 0.0% in the underweight group and 7.6% in the normal-weight group to 13.2% in the overweight group and 15.9% in the obesity group (*P* = 0.003).

Neonatal intensive care unit admission differed significantly across groups, with rates of 14.9%, 7.0%, 11.4%, and 17.5% in the underweight, normal-weight, overweight, and obesity groups, respectively (*P* = 0.010). Fetal asphyxia and neonatal sex distribution did not differ significantly across BMI categories (Table 2).

### Factors associated with adverse perinatal outcomes

In the multivariable logistic regression model using normal-weight women as the reference group, overweight and obesity before pregnancy were associated with higher odds of adverse perinatal outcomes. Compared with the normal-weight group, the adjusted OR was 1.38 for the overweight group (95% CI 1.01–1.90, *P* = 0.041) and 1.86 for the obesity group (95% CI 1.24–2.78, *P* = 0.003). Underweight was associated with a higher point estimate, but the association was not statistically significant (adjusted OR 1.42, 95% CI 0.84–2.40, *P* = 0.187). Maternal age, mode of conception, and neonatal sex were not significantly associated with adverse perinatal outcomes. The Hosmer-Lemeshow goodness-of-fit test showed a χ^2^ of 6.84 (*P* = 0.554), and the model had an AUC of 0.67 (Table 3).

**TABLE 3:** Multivariable logistic regression model for adverse perinatal outcomes.

Variables	Adjusted OR	95% CI	*P*-value
Maternal age, per year	1.03	0.98–1.08	0.214
Underweight vs. normal-weight	1.42	0.84–2.40	0.187
Overweight vs. normal-weight	1.38	1.01–1.90	0.041
Obesity vs. normal-weight	1.86	1.24–2.78	0.003
Mode of conception, ART vs. natural conception	1.28	0.72–2.27	0.402
Male sex	0.98	0.72–1.34	0.902

Model goodness-of-fit: Hosmer-Lemeshow χ^2^ = 6.84, *P* = 0.554; AUC = 0.67. ART, assisted reproductive technology.

### Infant growth and obesity at 6 months corrected age

At 6 months corrected age, WHZ, WAZ, and BAZ differed significantly across maternal BMI categories. WHZ increased from −0.10 ± 0.55 in the underweight group to 0.16 ± 0.50 in the normal-weight group, 0.45 ± 0.81 in the overweight group, and 0.61 ± 0.95 in the obesity group (*P* < 0.001). WAZ increased from −0.12 ± 0.58 to 0.19 ± 0.59, 0.44 ± 0.79, and 0.57 ± 0.91 across the four groups (*P* < 0.001). BAZ also increased across BMI categories, from 0.08 ± 0.52 in the underweight group to 0.31 ± 0.53 in the normal-weight group, 0.42 ± 0.72 in the overweight group, and 0.84 ± 0.87 in the obesity group (*P* < 0.001).

Height-for-age z-score did not differ significantly across BMI categories (*P* = 0.224), suggesting that differences in infant growth were more closely related to weight and adiposity than to linear growth. Infant obesity was more frequent in infants born to mothers with higher BMI, with rates of 2.1%, 6.4%, 9.5%, and 12.7% across the underweight, normal-weight, overweight, and obesity groups, respectively; however, the overall difference was borderline and did not reach conventional statistical significance (*P* = 0.055). Neurodevelopmental delay did not differ significantly among groups (*P* = 0.384) (Table 4).

**TABLE 4:** Infant growth status at 6 months corrected age according to maternal pre-pregnancy BMI category.

Variables	Underweight group	Normal-weight group	Overweight group	Obesity group	*P*-value
Infants	*N* = 47	*N* = 314	*N* = 220	*N* = 126	
WHZ	−0.10 ± 0.55	0.16 ± 0.50	0.45 ± 0.81	0.61 ± 0.95	<0.001^a^
WAZ	−0.12 ± 0.58	0.19 ± 0.59	0.44 ± 0.79	0.57 ± 0.91	<0.001^a^
HAZ	0.12 ± 0.52	0.19 ± 0.48	0.24 ± 0.50	0.27 ± 0.59	0.224^a^
BAZ	0.08 ± 0.52	0.31 ± 0.53	0.42 ± 0.72	0.84 ± 0.87	<0.001^a^
Infant obesity	1 (2.1%)	20 (6.4%)	21 (9.5%)	16 (12.7%)	0.055^b^
Neurodevelopmental delay	1 (2.1%)	3 (1.0%)	3 (1.4%)	4 (3.2%)	0.384^b^

WHZ, weight-for-height z-score; WAZ, weight-for-age z-score; HAZ, height-for-age z-score; BAZ, BMI-for-age z-score.

^a^Average and standard deviation. One-way Analysis of Variance.

^b^Number (percentage). Fisher exact test.

### Factors associated with infant obesity at 6 months corrected age

In the multivariable logistic regression model for infant obesity at 6 months corrected age, maternal obesity before pregnancy was associated with higher odds of infant obesity compared with normal weight (adjusted OR 2.23, 95% CI 1.18–4.20, *P* = 0.014). The association for overweight was in the same direction but did not reach statistical significance (adjusted OR 1.58, 95% CI 0.87–2.87, *P* = 0.132). Underweight was not significantly associated with infant obesity (adjusted OR 0.39, 95% CI 0.05–2.98, *P* = 0.366).

Breastfeeding at 6 months corrected age was associated with lower odds of infant obesity (adjusted OR 0.65, 95% CI 0.39–0.94, *P* = 0.026). Maternal age, mode of conception, and neonatal sex were not significantly associated with infant obesity. The Hosmer-Lemeshow goodness-of-fit test showed a χ^2^ of 5.72 (*P* = 0.679), and the model had an AUC of 0.71 (Table 5).

**TABLE 5:** Multivariable logistic regression model for infant obesity at 6 months corrected age.

Variables	Adjusted OR	95% CI	*P*-value
Maternal age, per year	1.05	0.99–1.12	0.104
Underweight vs. normal-weight	0.39	0.05–2.98	0.366
Overweight vs. normal-weight	1.58	0.87–2.87	0.132
Obesity vs. normal-weight	2.23	1.18–4.20	0.014
Mode of conception, ART vs. natural conception	1.39	0.61–3.18	0.435
Breastfeeding at 6 months corrected age	0.65	0.39–0.94	0.026
Male sex	1.03	0.62–1.70	0.914

Model goodness-of-fit: Hosmer-Lemeshow χ^2^ = 5.72, *P* = 0.679; AUC = 0.71. ART, assisted reproductive technology.

## Discussion

This two-center retrospective cohort study evaluated the associations of maternal pre-pregnancy BMI with pregnancy complications, adverse perinatal outcomes, and early infant adiposity at 6 months corrected age. Overall, higher maternal pre-pregnancy BMI was associated with higher rates of GDM, GHD, postpartum hemorrhage, fetal overgrowth, NICU admission, and infant adiposity at 6 months corrected age.

A major finding of this study was that women with higher pre-pregnancy BMI had a less favorable metabolic and obstetric profile. HbA1c increased across BMI categories, and both GDM and GHD were more frequent in the obesity group than in the normal-weight group. These findings are consistent with previous cohort studies and reviews showing that maternal overweight and obesity are associated with increased risks of GDM, hypertensive disorders, and other adverse maternal outcomes (2–6, 9–15). Mechanistically, excess maternal adiposity may promote insulin resistance, chronic inflammation, oxidative stress, endothelial dysfunction, altered placental function, and changes in maternal and neonatal metabolic or immune profiles, which together contribute to pregnancy-related metabolic and vascular complications (16–22, 33, 34).

Regarding neonatal outcomes, infants born to mothers with higher pre-pregnancy BMI had higher birthweight and higher rates of macrosomia. These results are biologically plausible and consistent with prior evidence linking maternal obesity, maternal hyperglycemia, altered lipid metabolism, placental changes, and fetal overgrowth (16, 33, 35). Maternal adiposity and related metabolic disturbance may increase maternal glucose and lipid availability, influence placental nutrient transport, and promote fetal insulin secretion, thereby contributing to fetal overgrowth. The underweight group showed the lowest mean birthweight and no cases of macrosomia, while FGR and NICU admission were numerically higher than in the normal-weight group. However, these differences should be interpreted cautiously because of the small number of underweight women.

At 6 months corrected age, infants born to mothers with higher pre-pregnancy BMI had higher WHZ, WAZ, and BAZ, whereas HAZ did not differ significantly across groups. This pattern suggests that maternal BMI was more strongly associated with infant weight and adiposity than with linear growth. These findings are consistent with the developmental origins of health and disease framework, which proposes that intrauterine metabolic exposure can shape offspring growth and metabolic risk (23).

The model for infant obesity showed that maternal obesity remained associated with infant obesity after adjustment for maternal age, mode of conception, breastfeeding, and neonatal sex, suggesting that pre-pregnancy adiposity may contribute to early infant adiposity through pathways not fully captured by postnatal feeding status (16, 21, 35). Moreover, breastfeeding was associated with lower odds of infant obesity at 6 months corrected age. This finding is consistent with evidence suggesting that breastfeeding may reduce the risk of childhood overweight and obesity (26–28). Breastfeeding may influence infant appetite regulation, gut microbiota development, metabolic signaling, and growth velocity. From a public health perspective, breastfeeding support may represent a feasible postnatal strategy to reduce early adiposity risk among infants exposed to maternal metabolic risk.

This study has several strengths. It included a two-center cohort of 707 mother-infant dyads, assessed infant anthropometry using WHO growth standard z-scores, and incorporated breastfeeding status in both the infant obesity model and descriptive analyses, representing a relatively comprehensive study design.

Several limitations should be considered. First, the retrospective design may be subject to residual confounding from unmeasured factors, such as dietary intake, physical activity, socioeconomic status, paternal BMI, maternal lipid profiles, and detailed infant feeding patterns. Second, pre-pregnancy weight was self-reported, which may have introduced BMI misclassification. Third, breastfeeding was defined as any breastfeeding at the 6-month corrected-age visit, without information on exclusivity, duration, or complementary feeding. Fourth, some outcomes, including ICP and fetal asphyxia, had few events, and the underweight group was relatively small, which may reduce the precision of estimates. Finally, follow-up was limited to 6 months corrected age, and longer-term studies are needed to determine whether early adiposity differences persist into childhood.

## Conclusion

In this two-center retrospective cohort study, higher maternal pre-pregnancy BMI was associated with increased rates of GDM, GHD, postpartum hemorrhage, macrosomia, NICU admission, and greater infant adiposity at 6 months corrected age. Compared with normal weight, maternal overweight and obesity were associated with higher odds of adverse perinatal outcomes, and pre-pregnancy obesity was further associated with higher odds of infant obesity at 6 months corrected age. Breastfeeding was associated with lower odds of infant obesity. These findings support preconception weight optimization, individualized gestational weight counseling, close monitoring and management of obesity-related pregnancy complications, and breastfeeding support to reduce early-life metabolic risk.

## Data Availability

The original contributions presented in this study are included in the article/supplementary material, further inquiries can be directed to the corresponding authors.
